# Pioneering Use of Extracorporeal Life Support in Intraoperative Cardiac Arrest in Dubai: A Case Report

**DOI:** 10.7759/cureus.76523

**Published:** 2024-12-28

**Authors:** Strahil N Kotsev, Nadine B Nour, Muhammad Sharif Allah Dad

**Affiliations:** 1 Department of Anaesthesia and Intensive Care, Latifa Hospital, Dubai Health, Dubai, ARE

**Keywords:** extracorporeal life support (ecls), inherited channelopathy, intra-operative cardiac arrest, team performance, va-ecmo

## Abstract

We describe, to our knowledge, the first use in Dubai of extracorporeal life support (ECLS) in a patient who suffered intraoperative cardiac arrest due to presumed cardiac channelopathy. A 40-year-old patient presented for open myomectomy surgery. She had no other medical problems apart from obesity. The patient denied any family history of surgery or anesthesia-related complications. Her initial electrocardiogram (ECG) and laboratory results were within reference limits. Intraoperatively, the patient suffered sudden cardiac arrest, from which she was resuscitated. Point-of-care cardiac ultrasound and intensive care unit (ICU)-performed echocardiography revealed severely reduced left ventricular contractility. Veno-arterial extracorporeal membrane oxygenation (V-A ECMO) and intra-aortic balloon pump were utilized in the immediate postoperative period. Although the patient's heart sustained more than 300 DC shocks, it recovered. Family members revealed that the patient's brother and sister had died in their 20s from sudden cardiac death. Another sister had been resuscitated a few years ago from intraoperative cardiac arrest, too. The case illustrates the importance of the patient's disclosure of relevant information. It supports the notion that ECLS can be used in the immediate postoperative period if surgical hemostasis is achieved. Controversies, such as the use of heparinization and the risk of bleeding, transthoracic echocardiography (TTE) versus transesophageal echocardiography (TEE), timely insertion of intracardiac defibrillator, and genetic screening, are discussed.

A learning point is that clinicians do not work in a vacuum. Organizational leadership can greatly impact outcomes, creating conditions for safer patient care.

## Introduction

Cardiac channelopathies are a rare cause of intraoperative drug-resistant arrhythmia and cardiac arrest [1]. Extracorporeal life support (ECLS) for refractory cardiac arrest has been studied [2,3].

We describe, to our knowledge, the first use of ECLS in such a case in Dubai. Controversies, such as the use of heparinization and the risk of bleeding, transthoracic echocardiography (TTE) versus transesophageal echocardiography (TEE), timely insertion of an intracardiac defibrillator, genetic testing, etc., are discussed. Time-pressured decision-making is described.

## Case presentation

A 40-year-old patient was admitted to a hospital in Dubai for elective open myomectomy. The history and physical revealed no medical problems apart from obesity (BMI 32). The patient did not disclose any family history of anesthesia or surgery-related problems. The electrocardiogram (ECG) (performed because of the patient's obesity) was normal.

Prior to the induction of anesthesia, a few premature ventricular contractions (PVCs) were noted on the monitor and reported to the team. As they were less than 5/min and of no hemodynamic significance, it was decided to proceed. The surgeons were asked to avoid the use of vasopressin for the reduction of surgical bleeding. Instead, they applied a uterine tourniquet.

After the removal of several fibroids, a short run of ventricular tachycardia (V-Tach) was noticed on the monitor, which reverted spontaneously. The team was informed, and help was summoned. An arterial line and central venous catheter were inserted. A crash trolley was fetched, and defibrillator pads were applied to the patient. A few minutes later, the patient went into ventricular fibrillation (V-Fib). She responded immediately to a DC shock. While the surgeons were closing the abdomen, the patient had several episodes of V-Fib. Advanced cardiac life support (ACLS) was provided. Most of the time, the patient responded to DC shocks with the return of spontaneous circulation without chest compressions. Amiodarone bolus and infusion were given, along with magnesium, but they failed to abort the episodes of V-Fib. The periods of chest compressions were few and short. Due to unstable hemodynamics, an adrenaline infusion was started. Point-of-care cardiac ultrasound was performed repeatedly. The initial impression was of a dilated left ventricle (LV) with reduced contractility. There were no sonographic signs of right ventricular strain or deep vein thrombosis to suggest pulmonary embolism. Volume status and responsiveness were periodically assessed, and pneumothorax was excluded by ultrasound and clinically. Electrolytes and temperature were normal. During the ACLS, a subcostal view of the heart was obtained, trying not to disrupt or delay the resuscitation efforts. The surgery was completed quickly, with approximately 200 mL of blood loss and adequate urine output. The patient was transferred to the intensive care unit (ICU), intubated, in sinus rhythm, and on adrenaline infusion. In spite of several shocks, the initial troponin level was normal, but the N-terminal pro B-type natriuretic peptide (NT-pro BNP) increased. Thyroid function tests and all other laboratory results were grossly normal, as was the chest X-ray (Table 1).

**Table 1:** Relevant laboratory results NT-ProBNP: N-terminal pro B-type natriuretic peptide

Component	Result	Reference range	Units
Sodium	136	136-145	mmol/L
Potassium	3.5	3.3-4.8	mmol/L
Chloride	104	98-108	mmol/L
Phosphate	2.3	2.7-4.5	mg/dL
Magnesium	2.24	1.6-2.6	mg/dL
Calcium	9	8.9-10.2	mg/dL
Urea	22	12-40	mg/dL
Creatinine	0.75	0.5-0.9	mg/dL
Hemoglobin	11.8	12-15	g/dL
Procalcitonin	0.03	<0.05	ng/mL
C-reactive protein	3.5	<5	mg/L
Free T4	13.5	12-22	pmol/L
TSH	1.16	0.27-4.2	uIU/L
Troponin	4	<12	ng/L
NT-ProBNP	9759	<125	mcg/mL

**Figure 1:**
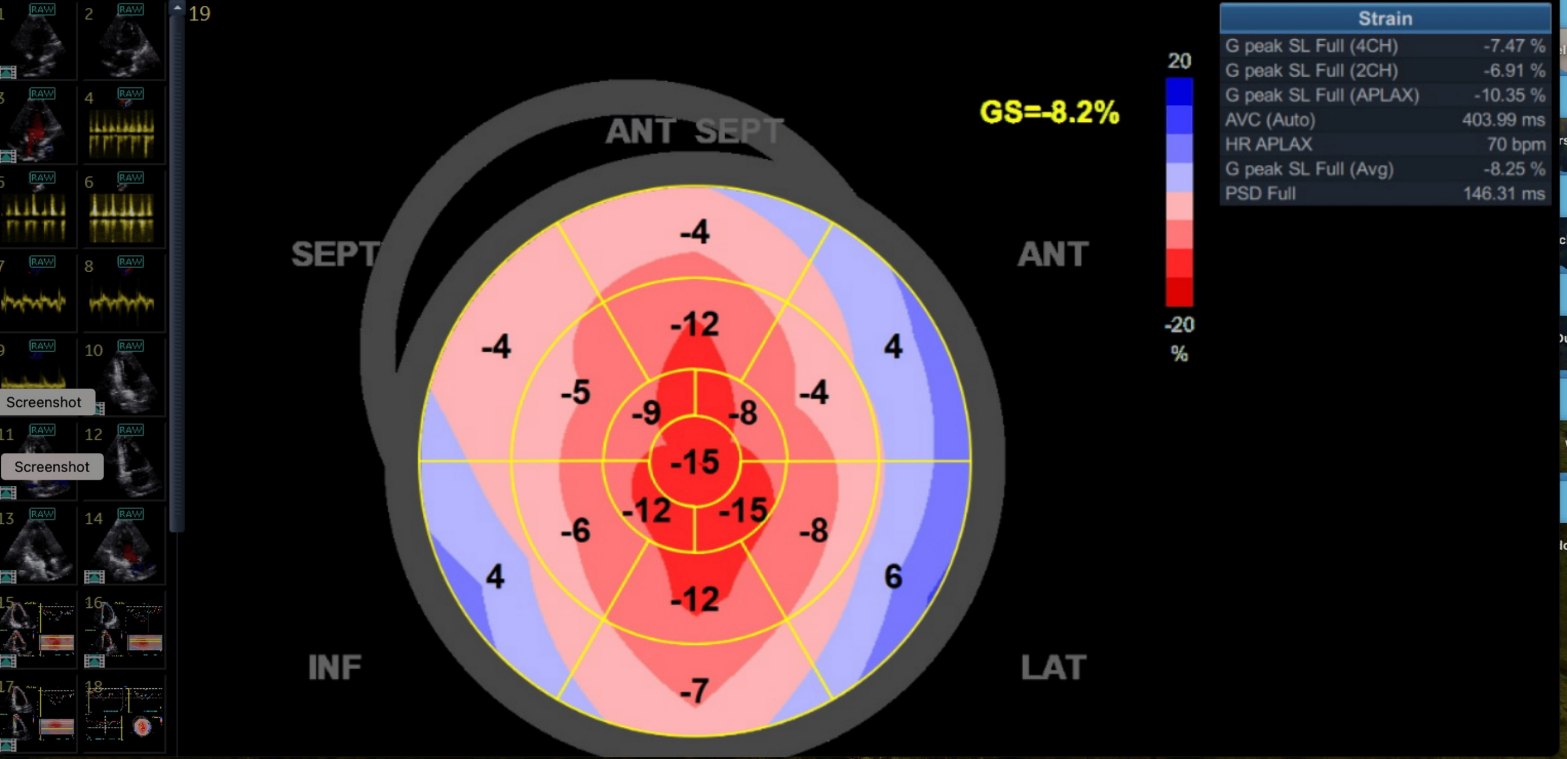
Global longitudinal strain postoperatively depicting severely reduced left ventricle contractility

In the ICU, the patient continued to have episodes of V-Fib, reverting back to sinus rhythm after each shock. Amiodarone, lignocaine, beta-blockers, mexiletine, and overdrive pacing failed to abort the episodes of V-Fib. A total of 252 shocks were delivered in less than a few hours. The adrenaline infusion was changed to noradrenaline, but the V-Fib episodes continued. A cardiology consult confirmed the initial findings of severely reduced LV contractility (Figure 1).

The ejection fraction was estimated at 25-30%, with average global longitudinal strain = -8% and LVOT VTI (left ventricular outflow tract velocity-time integral) 8.5 cm. The NT-proBNP was 78 times above the norm. There were no wall motion abnormalities (Video 1).

**Video 1:** Severely reduced left ventricle contractility on echo after ICU admission ICU: Intensive care unit

The extracorporeal membrane oxygenation (ECMO) team and intensivists were summoned. The patient was placed on veno-arterial extracorporeal membrane oxygenation (V-A ECMO). Later on, an intra-aortic balloon pump was added. During the insertion of the V-A ECMO, the patient continued to have episodes of V-Fib, reverting back to sinus rhythm after each shock without chest compressions. Due to the vast experience of Dubai's ECMO team, gathered during the COVID-19 pandemic, the procedure was completed within minutes [4].

The patient continued to have, for many days, episodes of V-Fib and V-Tach, reducing in frequency. She was successfully weaned off the V-A ECMO and intra-aortic balloon pump and extubated after a few days. Closure of the open abdominal wall (burst abdomen) was subsequently done under spinal anesthesia. She suffered another episode of intraoperative V-Tach, with restoration to sinus rhythm.

Family members disclosed that the patient's brother and sister died from sudden cardiac death in their 20s. Another sister was resuscitated several years ago from perioperative cardiac arrest.

**Figure 2:**
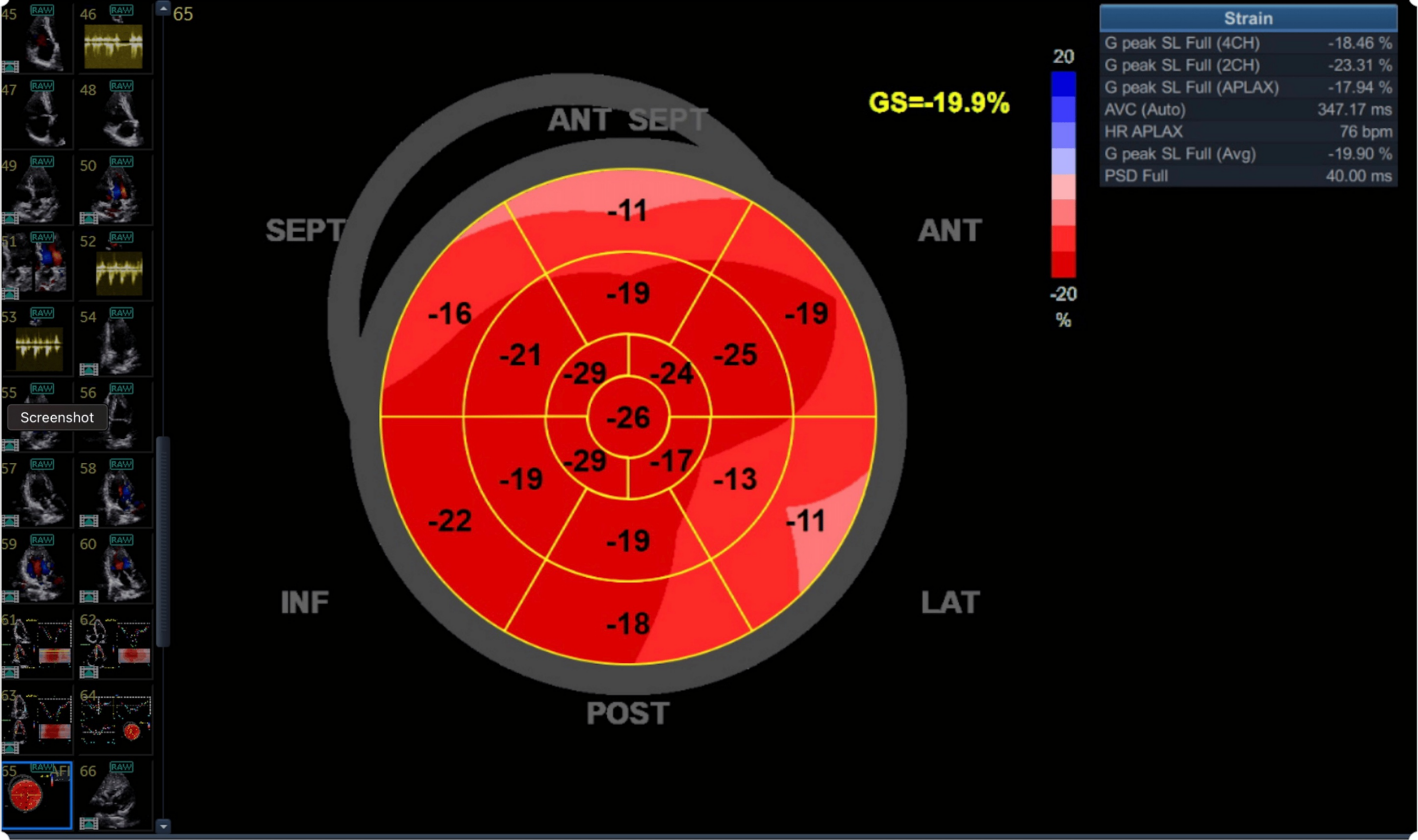
Global longitudinal strain depicting recovering of left ventricle contractility

Multiple ECGs done during the course of the events were normal. There was no prolongation of the QT interval or QTc. The electrophysiologists ruled out Brugada syndrome. Serial ECGs were done. After the initial reduction in contractility, the LV returned to normal (Figure 2).

For more than three weeks, the patient continued to have at least two episodes per day of V-Fib or V-Tach, reverting back to sinus rhythm after each shock without the need for chest compressions. Metanephrines drawn during her hospital stay were elevated. The patient suffered rhabdomyolysis and acute kidney injury, from which she recovered without dialysis.

After a month with daily episodes of V-Fib and shocks, the electrophysiologists inserted an internal cardiac defibrillator (ICD; Boston Scientific, Marlborough, MA, USA). The patient was discharged home. Family screening for cardiac channelopathy was planned.

**Figure 3:**
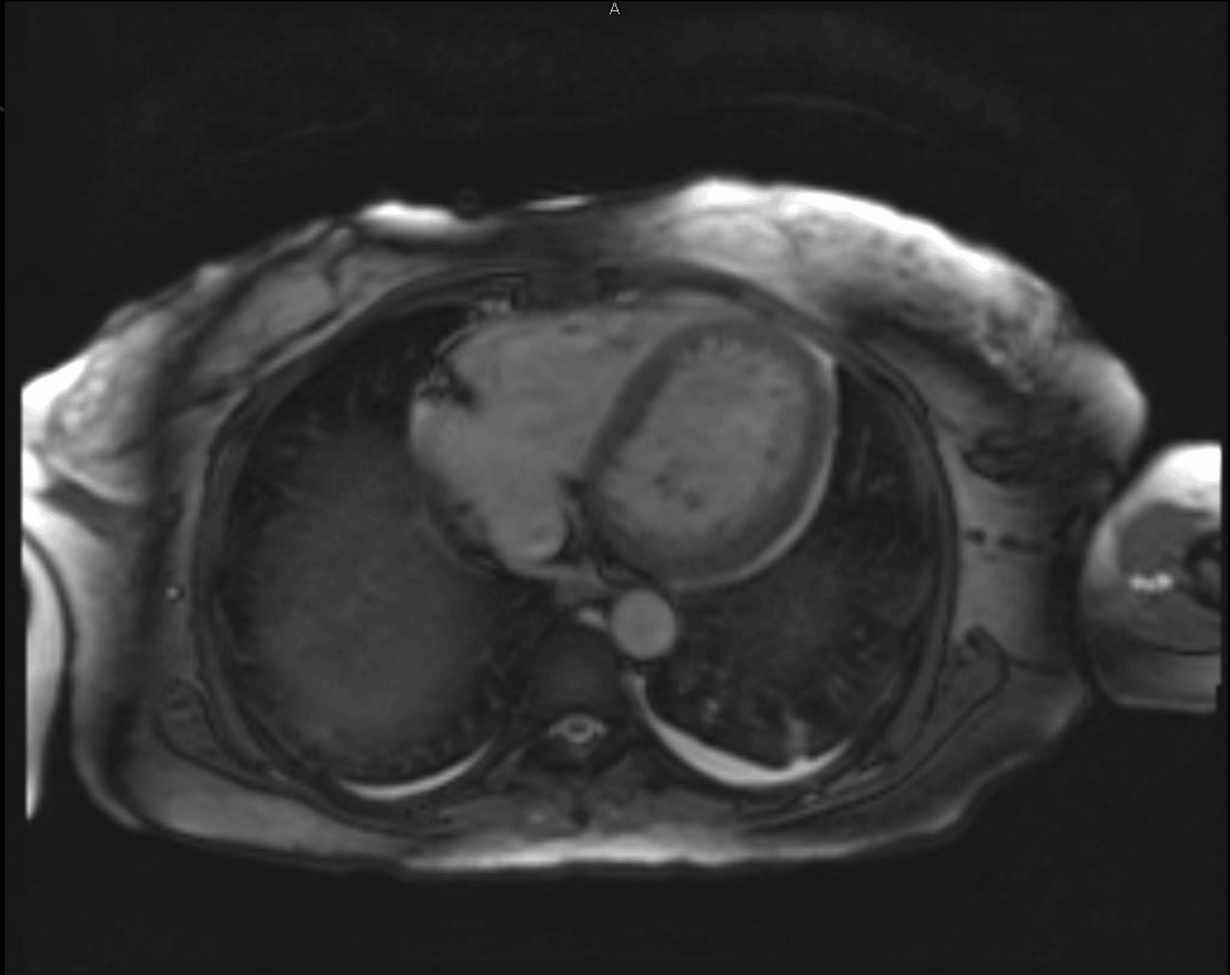
Cardiac MRI ruled out cardiomyopathy MRI: Magnetic resonance imaging

Two months after discharge, the patient is in sinus rhythm with no episodes of V-Fib or V-Tach. She is on amiodarone and bisoprolol. Cardiac magnetic resonance imaging (MRI) with contrast (cardiomyopathy protocol), done after resolution of the acute kidney injury, was grossly normal (Figure 3).

## Discussion

A point of discussion is heparinization for ECMO immediately after surgery. While there is a risk of bleeding, we believe the decision must be made on a case-by-case basis [5]. In our case, the surgeons were confident in the hemostasis. The latter allowed the ECMO team to proceed.

A controversy deserving discussion is TTE versus TEE in cardiac arrest. Although there might be delays in ACLS due to TTE, much depends on the team performance, timing of TTE, views obtained, and skills of the sonographer [6]. Timely management of subcostal views does not necessarily interfere with ACLS. The TEE interferes less with chest compressions, can guide the position for it (LVOT), and might be hands-free [7]. The transthoracic probe in our case was moved between the episodes of V-Fib over the chest wall to rule out pneumothorax. We did not have a TEE probe available.

Another point of discussion is how logistics and processes can impact patient care. The women's hospital where the arrest happened, although the biggest one in Dubai, did not have a computed tomography scanner or MRI. The cardiac center in Dubai, where the patient had been initially transferred on V-A ECMO, did not have an electrophysiology unit. The latter was in the trauma center. The above necessitated risky transfers between three hospitals.

It may have been better for the implantable cardioverter defibrillator (ICD) to be inserted earlier. Our initial impression of cardiomyopathy can be explained by the fact that, after multiple shocks, the myocardium can be stunned, and the ECG findings might resemble cardiomyopathy [8]. The patient's heart recovered from severely impaired to nearly normal. The cardiac MRI found no evidence of cardiomyopathy or thrombus, and normal LV and RV function.

Although the electrophysiologists ruled out Brugada syndrome, clinical criteria were present, and the patient was at the most affected age. ECG in Brugada's patients can change over time and can even be normal [9]. The same is true for long QT syndrome [10].

Stellate ganglion block has been described for the management of electrical storms. We did not try it, as the evidence is fringy, and we did not have experience with it [11].

Although planned, the genetic testing will be difficult, and the results might be inconclusive. Patients with the same genetic sequence often have incomplete penetrance [12].

## Conclusions

Cardiac channelopathies are difficult to recognize and manage. Disclosure of sudden cardiac death or arrest in relatives, from a patient or family, might be a clue. In a case of peri-operative cardiac arrest, decision-making is critical. Time-sensitive decisions might need to be made with insufficient information. The more exogenous the circumstances, the more important the coherence of a multidisciplinary team. Early involvement of electrophysiologists can bring valuable insights in such cases. The team must also have the resources to manage such patients. We believe a TEE probe and staff trained in its use must be available in every major hospital. The authors support the notion that, if the surgeons are confident in the hemostasis and an experienced ECMO team is available, ECLS is an option in the immediate postoperative period.

A more holistic view is that managerial decisions can greatly impact patient safety. Although Dubai hospitals have integrated electronic health records, echo lab images from one government hospital cannot be accessed from another or even from a different department in the same hospital. Opportunities for better integration do exist. The authors hope managerial decisions will be taken, solutions found, and eventually executed. It is necessary to eliminate, as much as possible, work in silos. Risky interhospital transfers must be avoided by system design. Clinicians must have timely access to important information through the integration of IT. All of the above will provide conditions for safer patient management. We have not discussed the psychological impact of such cases on the providers, but it is important to remember it. The clinical staff involved in such cases must not only be debriefed but supported, too.
